# Sweat Cortisol and Cortisone Determination in Healthy Adults: UHPLC-MS/MS Assay Validation and Clinical Application

**DOI:** 10.1155/2022/3133640

**Published:** 2022-11-29

**Authors:** Syed N. Alvi, Kafa Abuhdeeb, Muhammad M. Hammami

**Affiliations:** ^1^Clinical Studies and Empirical Ethics Department, King Faisal Specialist Hospital and Research Center, Riyadh, Saudi Arabia; ^2^Environmental Health Program, Department of Cell Biology, King Faisal Specialist Hospital and Research Center, Riyadh, Saudi Arabia

## Abstract

A simple and effective ultra-high-performance liquid chromatography assay linked to tandem mass spectrometry (UHPLC-MS/MS) for measuring cortisol and cortisone levels in human sweat has been developed and validated. A noninvasive world standard sweat collecting equipment was utilized to collect samples. The samples were analyzed using an Atlantis dC18 (2.1 × 100 mm, 3 *μ*m) column with a 2 mM ammonium acetate and acetonitrile (1 : 1, v : v) mobile phase. In an isocratic condition, the mobile phase was delivered at a flow rate of 0.3 ml/minute. A positive electrospray ionization interface with multiple-reaction monitoring mode was used to provide simultaneous quantification of cortisol, cortisone, and internal standard at transitions of 363.11 to 121.00, 361.18 to 163.11, and 367.19 to 121.24, respectively. The method was validated for cortisol and cortisone determination over a concentration range of 0.5–50 ng/mL The detection limits for cortisol and cortisone in human sweat were 0.3 and 0.2 ng/ml, respectively. The interday coefficients of variation of cortisol and cortisone were ≤8.5% and ≤10.01%, whereas bias was in the range from −7.9% to 2.1% and from −4.3% to 3.0%, respectively. The assay was successfully applied to evaluate the cortisol-to-cortisone ratio in sweat samples collected from healthy adult volunteers.

## 1. Introduction

Cortisol, a naturally occurring steroid, has an impact on a variety of body processes, including electrolyte balance, brain function, immune response, cardiovascular function, and metabolic activities [1, 2]. The 11ß-hydroxysteroidhydrogenase-2 (11ß-HSD2) isoenzyme, which interconverts biologically active cortisol to inactive cortisone, regulates cortisol metabolism. Monitoring the cortisol-cortisone ratio is considered the best indicator for treating people with adrenal diseases [3]. In routine practice, cortisol levels are assessed in serum, urine, and saliva to detect glucocorticoid-related diseases. These fluids are invasively sampled or have reliability issues that could produce inaccurate results. The detection of sweat cortisol is becoming more popular as a promising technique for noninvasive stress analysis [4].

Cortisol levels have been measured in biological fluids using a variety of methods, including chemiluminescence immunoassays (CLIA) [5], radioimmunoassays (RIA) [6], enzyme-linked immunosorbent assays (ELISA) [7], gas chromatography-mass spectrometry (GC-MS) [8], high-performance liquid chromatography (HPLC) [9], and liquid chromatography-tandem mass spectrometry (LC-MS) [10]. LC-MS/MS is the most popular technique for concurrently determining the cortisol-to-cortisone ratio in human serum/plasma [11], urine [12], saliva [13], and hair [14]. Few methods have been reported specifically for determining sweat cortisol levels [15]. However, no technique has been described for the simultaneous detection of cortisol and cortisone levels in sweat samples.

In this paper, we present a straightforward, confirmed UHPLC-MS/MS method for simultaneously assessing cortisol and cortisone in sweat. A macroduct sweat collection device, a popular noninvasive technique, is used to collect sweat. As a benchmark, the cortisol-cortisone ratio was computed using samples from healthy adult volunteers.

## 2. Methodology

### 2.1. Reagents and Chemicals

Cortisol (hydrocortisone) and cortisone were purchased from Acros Organics, NJ, USA. Cortisol-d4 was obtained from Sigma-Aldrich, MO, USA. Ammonium acetate, potassium phosphate (monobasic), sodium phosphate (dibasic), potassium chloride, sodium chloride, and acetonitrile (HPLC grade) were all provided by Fisher Scientific, NJ, USA. Milli-Q water was prepared by passing distilled water through the Milli-Q System (Millipore, Bedford, MA, USA). We prepared phosphate-buffered saline (PBS) by combining 0.01 M sodium phosphate, 0.0018 M potassium phosphate, 0.137 M sodium chloride, and 0.0027 M potassium chloride (pH = 7.4, adjusted with HCl). The study was approved by the Research Ethics Committee of King Faisal Specialist Hospital and Research Center, Riyadh, Saudi Arabia, under RAC No. 2191297.

### 2.2. Instrument and Analytical Conditions

An atmospheric pressure ionization (API) interface, an Acquity UHPLC with integrated solvent and sample management, and a tandem mass spectrometer (MS/MS) Xevo-TQD were all used in the investigation (Waters Corporation, Milford, MA, USA). The analytes were estimated using the Atlantis dC18 (2.1 × 100 mm, 3 *μ*m) steel column, which was shielded by an in-line filter. The mobile phase, which was a 50 : 50 (v/v) mixture of acetonitrile and 2.0 mM ammonium acetate, was filtered through a supor membrane filter before being delivered (0.3 mL/minute) (Pall Gelman Laboratory, MI, USA). The electrospray ionization source was used to operate the mass spectrometer instrument in a positive ion mode (capillary voltage, 1.50 kV; cone voltage, 35 volts). While nitrogen (1000 L/hr) was utilized for nebulization and desolvation, argon was employed for collision (3.6 × 10^−3^ mbar). The optimum collision energy for cortisol, cortisone, and the IS was found to be 20 eV. The ion source and desolvation temperatures were kept at 150°C and 500°C, respectively. Multiple-reaction monitoring (MRM) was used to detect cortisol, cortisone, and IS in positive ion mode at mass-to-charge transitions (*m*/*z*) of 363.11 to 121.00, 361.18 to 163.111, and 367.19 to 121.24 for cortisol, cortisone, and IS, respectively.

### 2.3. Preparation of Standard and Control Solutions

Stock solutions of cortisol, cortisone, and IS (1.0 mg/mL) were produced in methanol. Three quality control solutions (1.5, 25, and 45 ng/mL) and nine calibration standards with concentrations ranging from 0.5 to 50 ng/mL were prepared using PBS. We prepared three quality control samples by spiking an appropriate amount of cortisol and cortisone into human sweat (blank) to verify the usage of the calibration curve created in PBS. Cortisol and cortisone levels recorded between blank and spiked perspiration were compared to the anticipated rise level from the amount spiked (as evaluated against calibration standards). An IS working solution (100 ng/mL) in methanol was used in sample preparation. All stock and working solutions were kept at a temperature of −20°C and 4°C, respectively.

### 2.4. Sweat Sample Collection

A macroduct sweat collecting system (Model 3700) from France was used in this study. Sweat samples were collected in a unique disposable plastic container with a shallow concave underside, holding between 50 and 80 *μ*L. The macroduct is securely fastened to the frontal skin area of the mid-arm that has been iontophoretically stimulated. Hydraulic pressure forces perspiration from the sweat glands out of the ducts and into the microbore tubing spiral that runs between the skin and the concave underside of the macroduct collector. The total amount of perspiration can be easily measured using a small amount of blue dye. The amount of sweat produced varies greatly from person to person. However, an average of 60 microliters of sweat was collected over 30–40 minutes. The collected samples were transferred into a 200 *μ*L glass insert and kept at −20°C until they were examined.

### 2.5. Preparation of Samples

Sweat samples were taken out of the freezer and placed on a bench for 30 minutes to warm up. Then, 50 *μ*L of calibration standard, control, or volunteer sweat samples was placed into 200 *μ*L glass inserts, followed by 20 *μ*L of internal standard (100 ng/mL), and vortexed and centrifuged for five minutes. Then, we injected 10 *μ*L of samples into the UHPLC-MS/MS system for analysis.

## 3. Results and Discussion

### 3.1. Identification of the Components

**Figure 1:**
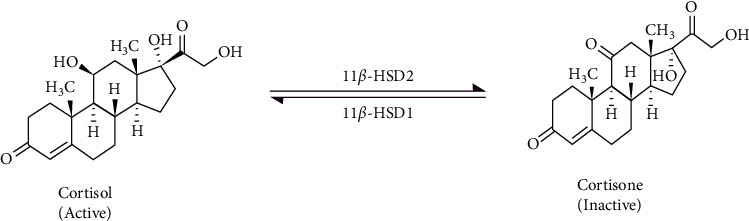
Interconversion of cortisol to cortisone via 11-ß-hydroxysteroid dehydrogenase type 2.

Figure 1 depicts the interconversion of biologically active cortisol to inert cortisone by the isoenzyme 11ß-hydroxysteroidhydrogenase-2. The precursor and product ions of cortisol and cortisone were determined along with the internal standard cortisol-d4 (internal standard). The standard mixture containing cortisol, cortisone, and cortisone-d4 at a concentration of 1 microgram each, using an infusing pump running at a flow rate of 20 *μ*L/minute. The mass spectrometry apparatus was set up using the IntelliStart software from Waters Corporation (Milford, MA, USA). We employed liquid chromatography and mass spectrometry to analyze saliva samples in settings similar to those previously described [16]. According to international reference standards, the method's linearity in the 0.5–50 ng/ml range, specificity, limit of detection and quantification, precision, and accuracy were all confirmed [17].

### 3.2. Effect of Matrix and Recovery

Peak areas of spiking sweat cortisol, cortisone, and IS at concentrations of 1.5, 25, and 45 ng/mL, respectively, and IS of 100 ng/mL were compared to equivalent peak areas of standards prepared in PBS by direct injection to assess matrix effect. However, no apparent matrix effect was observed. Cortisol and cortisone recovery were assessed by measuring cortisol and cortisone levels (1.5, 25, and 45 ng/mL) in sweat before and after addition. For cortisol and cortisone, the mean measured recovery was 92–102% and 92–104%, respectively.

### 3.3. Specificity

The method's specificity was tested by evaluating six different blank human sweat samples and six structurally related steroids that may be found in blank biological fluids, including testosterone, prednisolone, methylprednisolone, progesterone, 17-hydroxyprogesterone, and prednisone. All solutions were prepared in methanol: water (1 : 1, v : v) at a concentration of 1.0 *μ*g/mL, and 10 *μ*L was introduced into the system. The peaks of the analytes were found to be free of interference.

### 3.4. Linearity, Limit of Detection, and Quantification

Standard mixtures containing cortisol and cortisone in PBS at nine different concentrations (0.5–50 ng/ml) were evaluated to determine the assay's linearity. Regression analysis was used to examine the peak area ratios and the corresponding concentrations. Cortisol had a mean (*n* = 6) equation of *y* = 0.0543 × −0.0067, *r*^2^ = 0.9938, and cortisone had a mean (*n* = 6) equation of *y* = 0.0861 × −0.0035, *r*^2^ = 0.9900. The detection limits for cortisol and cortisone in human sweat were 0.3 and 0.2 ng/ml, respectively, whereas the quantification limit was 0.5 ng/ml.

### 3.5. Precision and Accuracy

Three quality control samples (1.5, 25, and 45 ng/ml) were examined for both intraday and interday precision and accuracy (bias). Cortisol's intraday (*n* = 10) and interday (*n* = 20) coefficients of variation (CV) varied in the range of 7.2% to 9.0%, and its bias varied in the range of -8.1% to 2.1%. Cortisone's intraday (*n* = 10) and interday (*n* = 20) CV varied in the range from 7.1% to 10.1%, whereas bias varied in the range from −7.6% to 3.0%, respectively (Table 1).

### 3.6. Application of the Method

**Figure 2:**
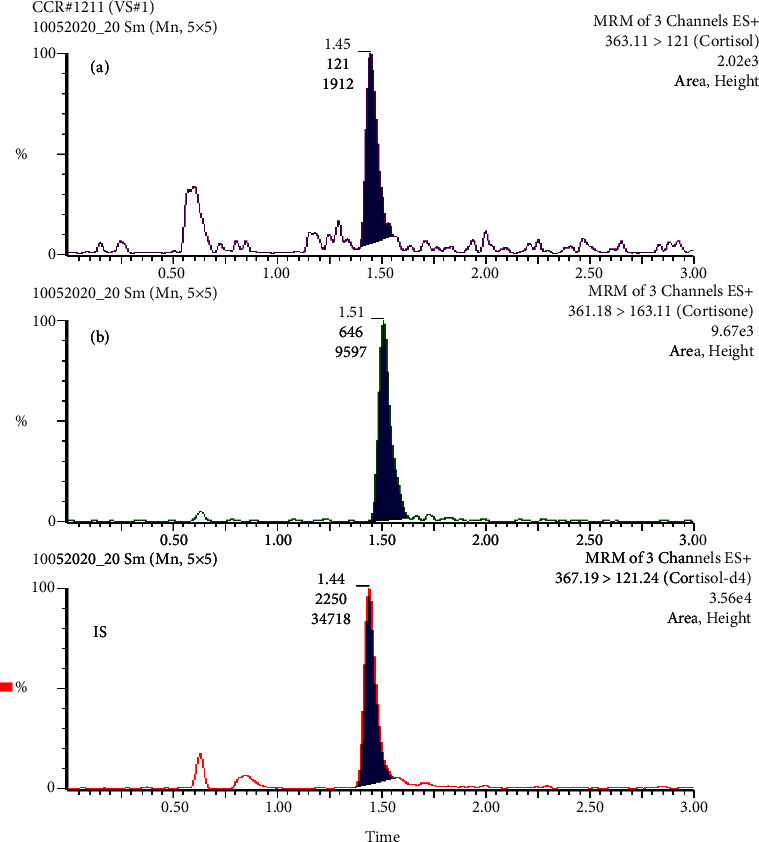
Multiple-reaction monitoring chromatogram of a sweat sample taken from a healthy volunteer spiked with IS (2.0 ng). The measured levels of (A) cortisol (1.8 ng/mL) and (B) cortisone (13.7 ng/mL).

**Figure 3:**
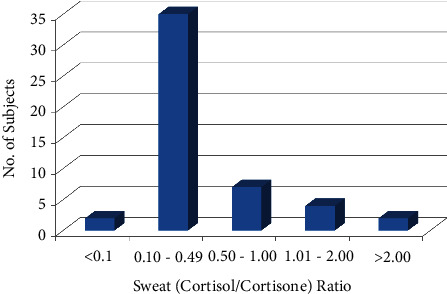
Sweat cortisol-cortisone ratio in healthy volunteer samples.

Figure 2 depicts a typical chromatogram of a sweat sample taken from a healthy subject, exhibiting cortisol and cortisone levels of 1.8 ng/ml and 13.7 ng/ml, respectively. A validated assay was successfully used to examine 50 sweat samples acquired from healthy subjects to establish a cortisol-cortisone reference interval in sweat.  Figure 3 shows the results. According to the findings, 46 out of 50 volunteers (92%) have a cortisol-cortisone ratio between 0.1 and 2.0. The cortisol-cortisone ratio reference range appears to be similar to that reported for the free cortisol-cortisone ratio in urine and saliva [12, 13].

## 4. Conclusion

In this article, a simple validated UHPLC-MS/MS assay for measuring cortisol and cortisone in human sweat has been described. The assay is based on an excellent noninvasive sweat collection standard device that has been successfully used to quantify both biochemical markers in human sweat samples.

## Figures and Tables

**Table 1:** Validation data.

Concentration (ng/mL)	Cortisol			Cortisone		
	1.5	25	45	1.5	25	45
*Intraday (n* *=* *10)*						
Mean (ng/mL)	1.45	22.98	42.27	1.39	23.96	46.69
SD	0.13	1.79	3.30	0.10	2.36	4.05
CV (%)	9.0	7.8	7.8	7.1	9.8	8.7
Bias (%)	−3.6	−8.1	−6.1	−7.6	−4.2	3.7
Recovery (%)	96	92	94	92	96	104

*Interday (n* *=* *20)*						
Mean (ng/mL)	1.53	23.04	42.57	1.44	23.93	46.33
SD	0.13	1.66	3.30	0.11	2.42	4.12
CV (%)	8.5	7.2	7.8	7.7	10.1	8.9
Bias (%)	2.1	−7.9	−6.1	−4.3	−4.3	3.0
Recovery (%)	102	92	94	96	96	103

The SD stands for standard deviation. The coefficient of variation (CV) is SD divided by the mean measured level, then multiplied by 100. Bias (%) = (measured level−nominal level/nominal level) × 100.

## Data Availability

The data used to support the findings of this study are available from the corresponding author upon request.
